# Long-term memory-based control of attention in multi-step tasks requires working memory: evidence from domain-specific interference

**DOI:** 10.3389/fpsyg.2014.00408

**Published:** 2014-05-09

**Authors:** Rebecca M. Foerster, Elena Carbone, Werner X. Schneider

**Affiliations:** ^1^Neuro-Cognitive Psychology, Department of Psychology, Bielefeld UniversityBielefeld, Germany; ^2^Cluster of Excellence ‘Cognitive Interaction Technology,’ Bielefeld UniversityBielefeld, Germany

**Keywords:** working memory, interference, attention, long-term memory, visuospatial, verbal, learning

## Abstract

Evidence for long-term memory (LTM)-based control of attention has been found during the execution of highly practiced multi-step tasks. However, does LTM directly control for attention or are working memory (WM) processes involved? In the present study, this question was investigated with a dual-task paradigm. Participants executed either a highly practiced visuospatial sensorimotor task (speed stacking) or a verbal task (high-speed poem reciting), while maintaining visuospatial or verbal information in WM. Results revealed unidirectional and domain-specific interference. Neither speed stacking nor high-speed poem reciting was influenced by WM retention. Stacking disrupted the retention of visuospatial locations, but did not modify memory performance of verbal material (letters). Reciting reduced the retention of verbal material substantially whereas it affected the memory performance of visuospatial locations to a smaller degree. We suggest that the selection of task-relevant information from LTM for the execution of overlearned multi-step tasks recruits domain-specific WM.

## Introduction

Humans can efficiently perform highly complex tasks every day without much effort. Examples are driving a bicycle or a car, reading a newspaper, or singing along a favorite song. The ease with which these tasks are performed should be due to a substantial long-term memory (LTM) contribution (e.g., Neumann, 1984, 1990; Logan, 1988, 1990).

Such highly LTM-controlled skills are often viewed as automatized. Theories of automatization and skill proceduralization claim that automatized processes are executed without requiring any attention or WM resources. According to the two-process theory of information processing (Schneider and Shiffrin, 1977a,b), automatic processes do not need attention or conscious control and can be performed interference-free in parallel with other processes. The concept of direct parameter specification (Neumann, 1984, 1990) postulates that relevant action parameters are specified directly via the conjunction of sensory input information and LTM-retrieved skill information. The instance theory of automatization (Logan, 1988, 1990) assumes that an automatized action is based on direct-access retrieval of the strongest associated LTM instance. Finally, researchers focusing on skill argue that procedural knowledge is not constantly consciously controlled and does not rely on WM (e.g., Fitts and Posner, 1967; Anderson, 1993; Beilock et al., 2002). Thus, LTM-based tasks are viewed to be automatized so that they do not involve attention and WM.

However, this theoretical sketch seems not to be as clear as traditionally thought. Recently, we investigated how LTM is involved in the control of attention and eye movements in a complex multi-step task (Foerster et al., 2011, 2012). In Foerster et al. (2011), participants were trained for 14 days in the high-speed sensorimotor task of speed stacking. In speed stacking, pyramids of plastic cups have to be stacked up and down as fast as possible in a predefined order. Eye movements—overt markers of visual attention (e.g., Deubel and Schneider, 1996)—were recorded and compared across the first and the last training day. With learning, participants became faster and performed fewer fixations. We suggested that the control of visual attention and eye movements becomes less sensory-based and more LTM-based during learning. This means that LTM sequentially guides attention and eye movements to task-relevant positions in the environment. This suggestion was further supported by the fact that the extensively trained participants performed a highly similar task-related sequence of eye movements when performing the task in complete darkness (Foerster et al., 2012). Therefore, attention is still required. However, the allocation of attention and eye movements during this well-practiced multi-step sensorimotor task in dark must be grounded in LTM. Does this imply that working memory (WM) processes are not involved? More precisely, does LTM directly control where to attend and where to look next or are respective target locations first activated in visual WM?

There are contradictory assumptions about the interplay of LTM and WM. Baddeley (2012), for instance, stated that the integration of perception, LTM, and action into the multi-component WM model is an important next step as it is not clear yet whether and how LTM-based tasks require WM processes. According to Baddeley (1986; Baddeley and Hitch, 1974) WM consists of multiple components for temporary storage and manipulation of limited information. One passive store, the articulatory loop, is concerned with verbal information. Another passive store, the visuospatial sketchpad, is concerned with visuospatial information. An active control system, the central executive, manipulates incoming and stored information. A fourth component—the episodic buffer—was added later (Baddeley, 2000). That is a multidimensional store receiving input from both the verbal and the visuospatial store. It is connected to LTM, and controlled by the central executive (Baddeley et al., 2011). Thus, one possibility how LTM might interact with WM is, that LTM content is activated by transferring it to the central executive which would result in global interference across LTM and WM tasks. Another possibility is that LTM content might be retrieved by activation in domain-specific WM stores resulting in domain-specific interference across LTM and WM tasks. Finally and suggested by theories of automatization (e.g., Schneider and Shiffrin, 1977a,b) and skill acquisition (e.g., Fitts and Posner, 1967; Anderson, 1993; Beilock et al., 2002), LTM information might directly control for action requiring neither the domain-specific store nor the central executive. This would result in completely interference-free dual-task performance of LTM and WM tasks.

Indeed, several investigations of well-learned multi-step tasks such as tea-making (Land et al., 1999), sandwich-making (Hayhoe et al., 2003), or car driving (Land and Tatler, 2001) indicated that humans make usually little use of their WM when engaged in these tasks (e.g., Droll et al., 2005). In contrast, visual information seems to be gathered just when it is needed—the so-called “just-in-time” strategy (Hayhoe et al., 2003). Evidence for the dissociation between WM and LTM also comes from laboratory tasks. Attention during visual-search tasks seems to be only influenced by WM items if the search target varies from trial to trial (e.g., Woodman et al., 2013). However, if the search target stays the same over several trials, WM maintenance and visual search do not interfere. Complementary, if the repeated targets are used as distractors in subsequent trials, performance is disturbed (Schneider and Shiffrin, 1977a,b; Kyllingsbaek et al., 2001). Woodman et al. (2013) suggest that in the case of constant search targets, LTM takes over in providing the search template. In summary, results from highly controlled laboratory tasks also argue for direct LTM-control of visual attention without the involvement of visual WM.

On the other hand, there is growing consensus that selective attention is strongly related to WM processes (e.g., Olivers et al., 2011). Selective visuospatial attention usually determines which information of the environment will access WM (Awh et al., 2006; Bundesen and Habekost, 2008). Not only encoding in WM but also WM maintenance has been linked to attention (e.g., Awh et al., 2006; Gazzaley and Nobre, 2012). It has been suggested that covert attention might be involved in visuospatial rehearsal. This assumption was supported by behavioral (e.g., Smyth and Scholey, 1994; Smyth, 1996; Awh et al., 1998; Theeuwes et al., 2011) and by neuroimaging evidence (e.g., Awh et al., 1995, 1999, 2000; Awh and Jonides, 2001). Finally, it has been postulated that attention helps retrieving information from WM (e.g., Johansson et al., 2011; Gazzaley and Nobre, 2012). Given this link between visual attention and the use of WM information (see also, Schneider, 2013), again the question emerges whether retrieving information from LTM for attentional control can bypass WM.

Our approach attempted to tackle this question on the basis of a dual-task paradigm that combines WM retention with the execution of a well-practiced multi-step task. More specifically, participants had to perform either a verbal task (high-speed poem reciting) or a sensorimotor task (speed stacking), while maintaining either verbal (letters) or visuospatial (locations) material in WM. We chose high-speed poem reciting (reciting a poem by heart as fast as possible) and speed stacking (stacking up and down cups as fast as possible) because both multi-step tasks can be learned easily and rapidly and provide short and comparable execution times.

Based on the considerations outlined above, two opposing predictions can be made. If LTM controls attention directly without the involvement of WM, no interference should arise between highly practiced multi-step tasks and WM-span tasks. If LTM-based control of attention requires WM, interference should occur. Such an interference could be either global or domain-specific in nature, i.e., interference effects could be observed either across or within information domain (verbal vs. visuospatial).

## Materials and methods

### Participants

Ten students from Bielefeld University, Germany, participated in the experiment. Seven of them took part in a speed-stacking automatization study (Foerster et al., 2011) and the other three participants ran through the same speed-stacking training before participating in the present experiment. Participants' age ranged from 21 to 32 years with a mean of 26. All participants had either normal or corrected-to-normal vision, were naive with respect to the aims of the study, and were paid for their participation. The study was performed in accordance with the ethical standards laid down in the 1964 Declaration of Helsinki. All participants gave their informed consent to be included in the study.

### Apparatus and stimuli

A notebook with a 15.4 inch screen, with a resolution of 1024 × 768 pixels and speed-stacking equipment (cups, timer, and mat) were used for the experiment. Participants were seated in front of the screen and the speed-stacking equipment was placed in-between them and the screen. The distance to the screen was approximately 60 cm. Stimulus presentation of the WM task was controlled by the Experiment Builder software (SR Research, Ontario, Canada). Stimuli were displayed on a black background. The verbal memory stimuli were yellow consonants (B, F, J, L, N, Q, R, V, and X), appearing successively inside of a white frame (subtending approximately 2.86° of visual angle) centered on the screen. For the visuospatial WM-span task, gray filled white squares (again subtending approximately 2.86° of visual angle) were distributed in a fixed layout across the screen, and individual frames successively changed their inner color to yellow and back to gray, in a random order. The visuospatial task was similar to the Corsi Block task of De Renzi and Nichelli (1975). Neither a letter nor a location was repeated within a sequence. The poem consisted of four quatrains with rhyming couplets and iamb as measure (see Appendix).

### Design

We analyzed the data with repeated measures analyses of variance. In case of significant effects, data was analyzed further with planned *t*-tests. The within-subject variables were WM-span task (none, verbal, and visuospatial) and multi-step task (none, reciting, and stacking). WM-span condition was blocked starting with a multi-step task without WM-span task (single-task condition) as a first block, and the multi-step tasks with verbal and visuospatial WM-span task (dual-task conditions) as second and third block. The order of blocks 2 and 3 was counterbalanced across participants. The multi-step task conditions were intermixed within the two latter WM-span blocks. The first block of the experiment (no WM-span task) consisted of six stacking and six reciting trials. Each of the other two WM-span blocks (verbal and visuospatial) consisted of 18 experimental trials, with six trials each for the three multi-step task conditions (none, reciting, and stacking), adding up to a total of 48 trials. Two practice trials (one verbal WM-span trial and one visuospatial WM-span trial, both without multi-step task) at the beginning of the second block were added to ensure that the participants followed the instruction.

The dependent variables were percentage correct for the WM-span tasks as well as completion time and error rate for the speed-stacking task and the poem-reciting task. WM-span performance was considered correct when all memory items were reported in the correct order. Respectively, speed stacking and poem reciting were considered correct when all actions and words were correct. The performance measure of the multi-step tasks was the duration of a complete stacking or reciting sequence. We defined a stacking error as one or more cups falling or sliding down. Skipping, substituting, adding, or transposing of one or more words was defined as a reciting error.

### Procedure

Each experimental manipulation was preceded by a speed-stacking and a poem-reciting training period as well as a refreshment day directly before the experimental day. Speed stacking consists of a fixed sequence of stacking up and down pyramids of plastic cups as fast as possible. Number, order, and direction of the stacking movements are predetermined (for an illustrative video visit http://www.speedstacks.com/about/history.php). The speed-stacking training phase consisted of 14 days with 45 min practice each day (details are reported in Foerster et al., 2011). The poem-reciting training lasted 50 min on a single day consisting of 10 min silent memorization and 40 min reciting at maximum speed. This poem-reciting training was preceded and followed by reading aloud the poem three times. On the refreshment day, both stacking, and reciting had to be performed as fast as possible for 30 min each.

The last day was the experimental day and started with the first block of high-speed stacking and high-speed poem reciting without parallel WM-span task. The instruction was again to perform as fast as possible. This initial calculation of the participants' performance in stacking and reciting served as a baseline for the multi-step tasks. The trial speed of both multi-step tasks was measured by a timer and then transferred and stored on the notebook. The accuracy was marked by the experimenter.

Afterwards, the dual-task trials started with a written instruction appearing on the screen. Each trial started with a left mouse button press followed by the sequence of memory items, either four consonants or three locations. This difference in number of to-be-remembered items was necessary to ensure equal task difficulty (see Results section). Each item was shown for 400 ms with an inter-stimulus interval (ISI) of 400 ms. Following the stimulus sequence, a written message was shown on the screen for 20 s informing the participants about the activity they had to accomplish within this delay (none, reciting, or stacking). A tone signaled the start and the end of the delay. Participants were instructed to be as accurately as possible in the memory task.

**Figure 1:**
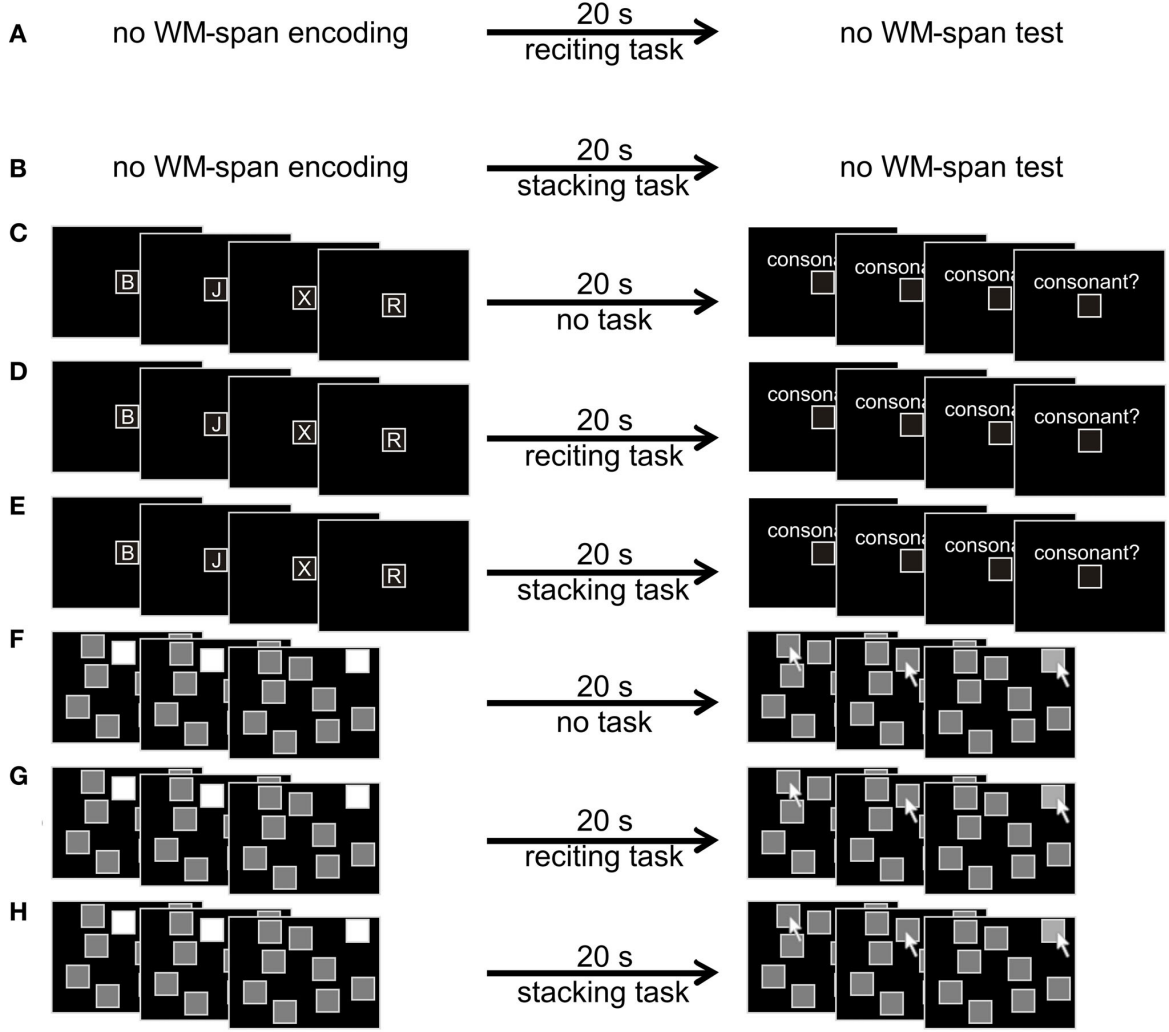
**Trial sequences for the eight different experimental combinations of conditions. (A)** No WM-span task with reciting as multi-step task. **(B)** No WM-span task with stacking as multi-step task. **(C)** Verbal WM-span task with reciting as multi-step task. **(D)** Verbal WM-span task with stacking as multi-step task. **(E)** Verbal WM-span task without multi-step task. **(F)** Visuospatial WM-span task with reciting as multi-step task. **(G)** Visuospatial WM-span task with stacking as multi-step task. **(H)** Visuospatial WM-span task without multi-step task. Consonants were typed in via the keyboard. Locations were clicked on with the mouse cursor.

For the verbal WM-span test, a central frame was shown on the screen and participants had to type in the letters in the correct order via the keyboard. Spatially distributed frames were shown on the screen for the visuospatial WM-span test, and participants had to select the locations via the mouse cursor in the correct order and confirm each selection with a left mouse click. The recording of the WM span stopped as soon as the participants had made an error or had reproduced the complete sequence correctly. The reproduction was followed by a feedback (“correct” or “incorrect”). Trial sequences for all six combinations of conditions are shown in Figure 1. The participants were supposed to memorize the items as accurately as possible and to stack and recite as fast as possible.

## Results

### Learning of speed stacking and poem reciting

**Figure 2:**
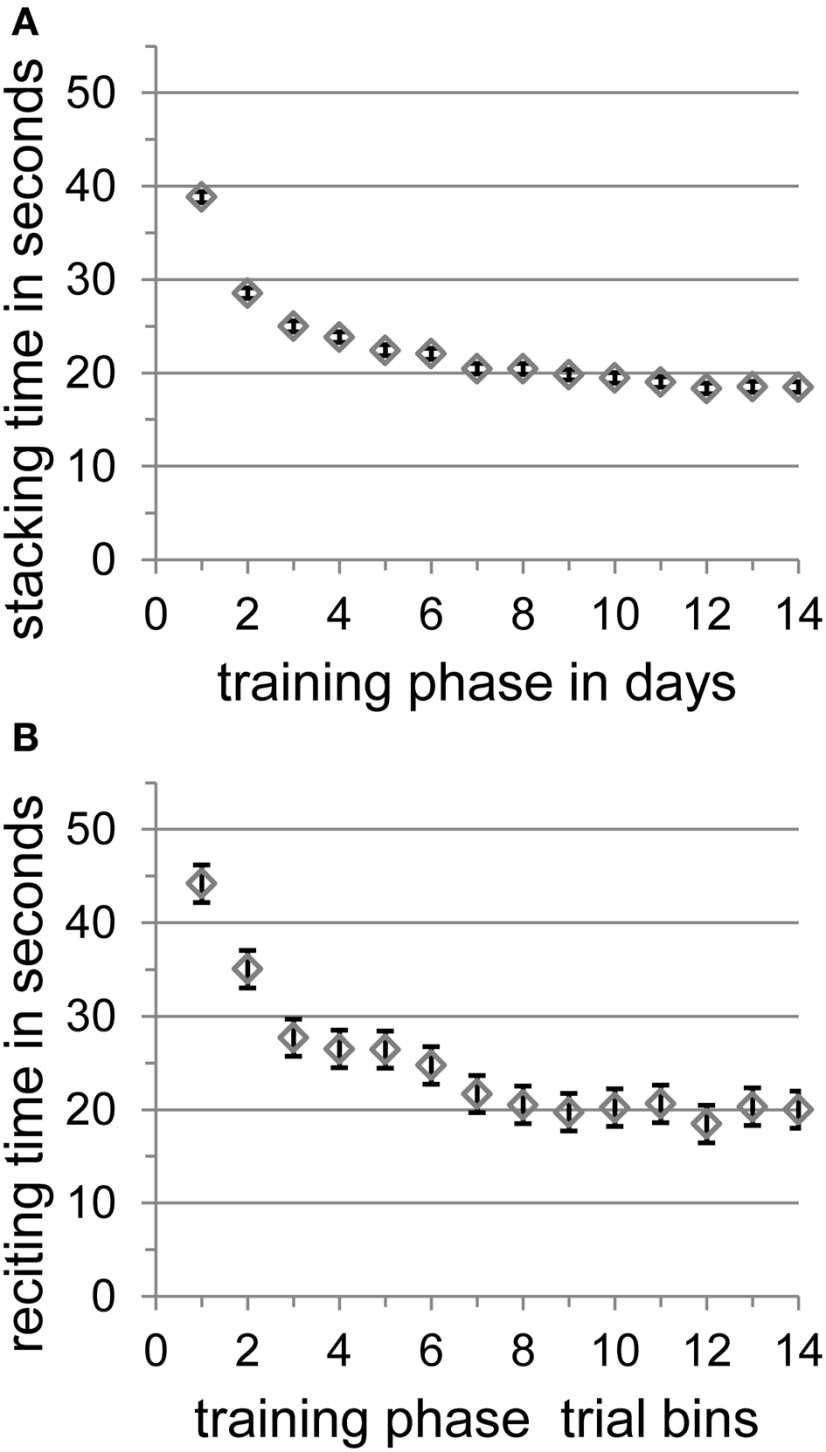
**(A)** The mean stacking time in seconds with standard error of the mean according to Loftus and Masson (1994) per training phase in days. **(B)** The training trials of each participant's reciting automatization day were split into 14 equal bins, and means of reciting times were calculated. The figure shows the mean reciting time in seconds with standard error of the mean according to Loftus and Masson (1994) per training phase in 14 trial bins.

Stacking time decreased significantly from the first (38.83 s) to the last (18.49 s) training day [*t*_(9)_ = 8.55, *SE* = 2.38, *p* < 0.001] and participants achieved a mean stacking time of 18.49 s with a mean best time of 12.63 s on the last training day. There was no further significant improvement from the 11th day on [day 11–14: *t*_(1, 9)_ = 2.00, *SE* = 0.85, *p* = 0.077; day 12–14: *t*_(1, 9)_ = 0.24, *SE* = 1.85, *p* = 0.815; day 13–14: *t*_(1, 9)_ = 0.37, *SE* = 0.72, *p* = 0.723]. Speed-stacking performance over training days is shown in Figure 2A.

Because the whole poem-reciting training took place on a single day, we split up the training trials of each participant into 14 equal bins and calculated means of reciting times for each bin. All participants learned the poem as reflected by the significant overall decrease of mean reciting time between the first bin (44.18 s) and the last bin (20.01 s) of all trials [*t*_(9)_ = 4.63, *SE* = 5.23, *p* < 0.01]. There was no further significant improvement from the 8th bin on [bin 8–14: *t*_(1, 9)_ = 0.51, *SE* = 0.98, *p* = 0.625; bin 9–14: *t*_(1, 9)_ = 0.35, *SE* = 0.80, *p* = 0.734; bin 10–14: *t*_(1, 9)_ = 0.10, *SE* = 2.02, *p* = 0.922; bin 11–14: *t*_(1, 9)_ = 0.67, *SE* = 0.93, *p* = 0.518; bin 12–14: *t*_(1, 9)_ = 0.99, *SE* = 1.55, *p* = 0.348; bin 13–14: *t*_(1, 9)_ = 0.18, *SE* = 1.79, *p* = 0.865]. Participants achieved a mean best reciting time of 14.47 s that corresponds to a reciting rate of seven syllables per second. This best reciting time did not differ significantly from the mean best reading time of 14.41 s (seven syllables per second) before training [*t*_(1, 9)_ = 2.24, *SE* = 1.31, *p* > 0.05] nor from the mean best reading time of 12.43 s (eight syllables per second) after training [*t*_(1, 9)_ = 1.84, *SE* = 1.11, *p* > 0.05]. Poem-reciting performance over training bins is shown in Figure 2B.

### Performance of multi-step tasks with concurrent WM-span tasks

**Figure 3:**
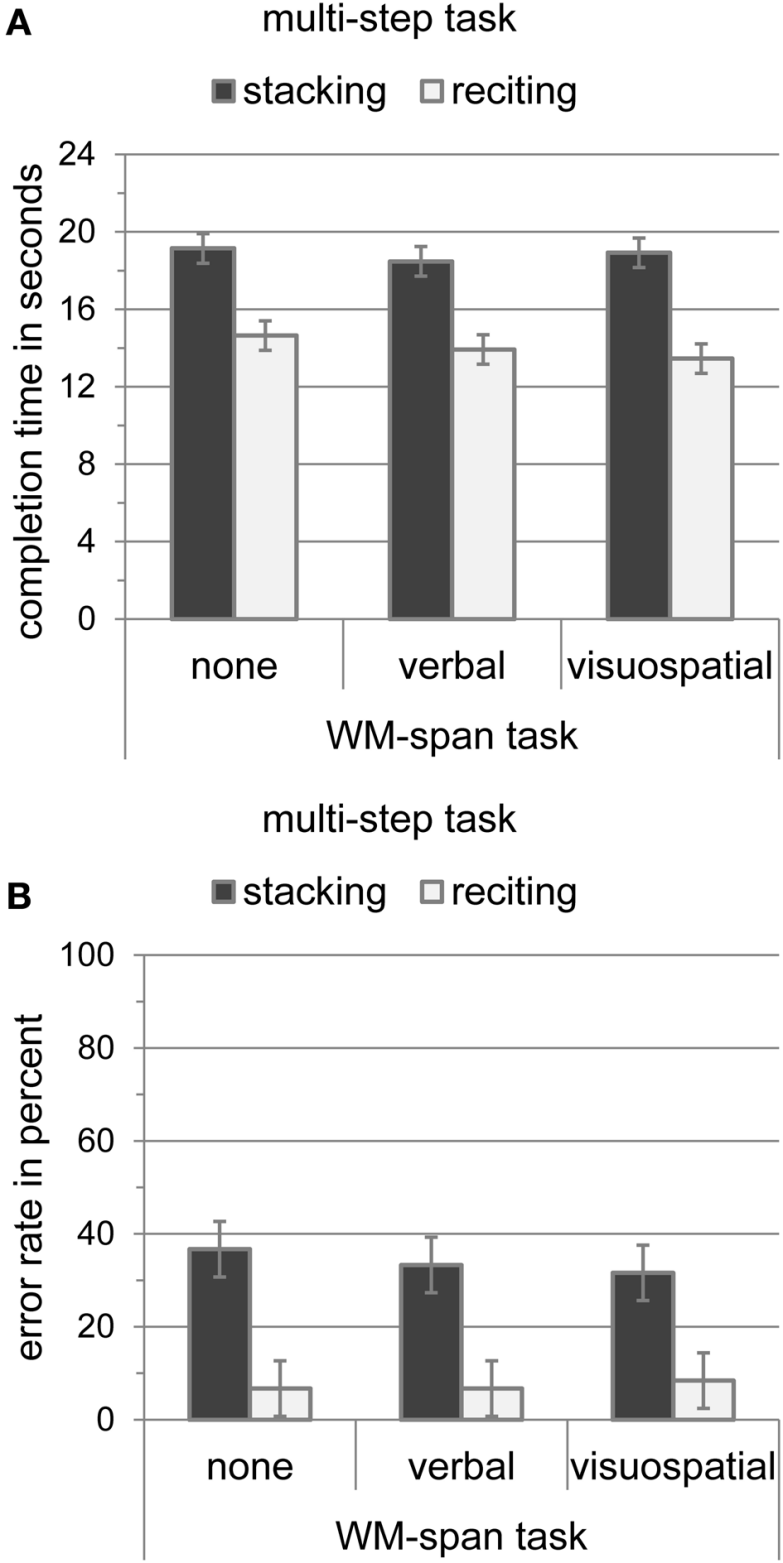
**(A)** Completion time of the multi-step tasks (stacking and reciting) in seconds with standard error of the mean according to Loftus and Masson (1994) during the WM-span tasks (none, verbal, and visuospatial). **(B)** Error rate of the multi-step tasks (stacking and reciting) in percent with standard error of the mean according to Loftus and Masson (1994) during the WM-span tasks (none, verbal, and visuospatial).

Stacking and reciting speed and accuracy are depicted in Figure 3. To test whether the WM-span tasks affected stacking or reciting performance, we conducted two 2 × 3 analyses of variance for task completion time and error rate as dependent variables with multi-step task (reciting and stacking) and WM-span task (none, verbal, and visuospatial) as within-subject variables. The analysis of task completion time revealed a significant main effect of multi-step task [*F*_(1, 9)_ = 14.07, *MSE* = 351.19, *p* < 0.01], indicating that participants could recite the poem faster (14.01 s) than they could stack the cups (18.85 s). Neither the main effect of WM-span task [*F*_(2, 18)_ = 2.36, *MSE* = 3.28, *p* > 0.05] nor the interaction of multi-step task and WM-span task [*F*_(2, 18)_ = 2.50, *MSE* = 1.47, *p* > 0.05] were significant. The analysis of error rate revealed a significant main effect of multi-step task [*F*_(1, 9)_ = 17.28, *MSE* = 1.06, *p* < 0.01], indicating that participants made less errors when reciting the poem (7.27%) than when stacking the cups (33.87%). Neither the main effect of WM-span task [*F*_(2, 18)_ = 0.11, *MSE* = 0.002, *p* > 0.05] nor the interaction of multi-step task and WM-span task [*F*_(2, 18)_ = 0.14, *MSE* = 0.01, *p* > 0.05] were significant. Results indicate that stacking and reciting performance are not influenced by simultaneous WM retention.

### Performance of WM-span tasks with concurrent multi-step tasks

**Figure 4:**
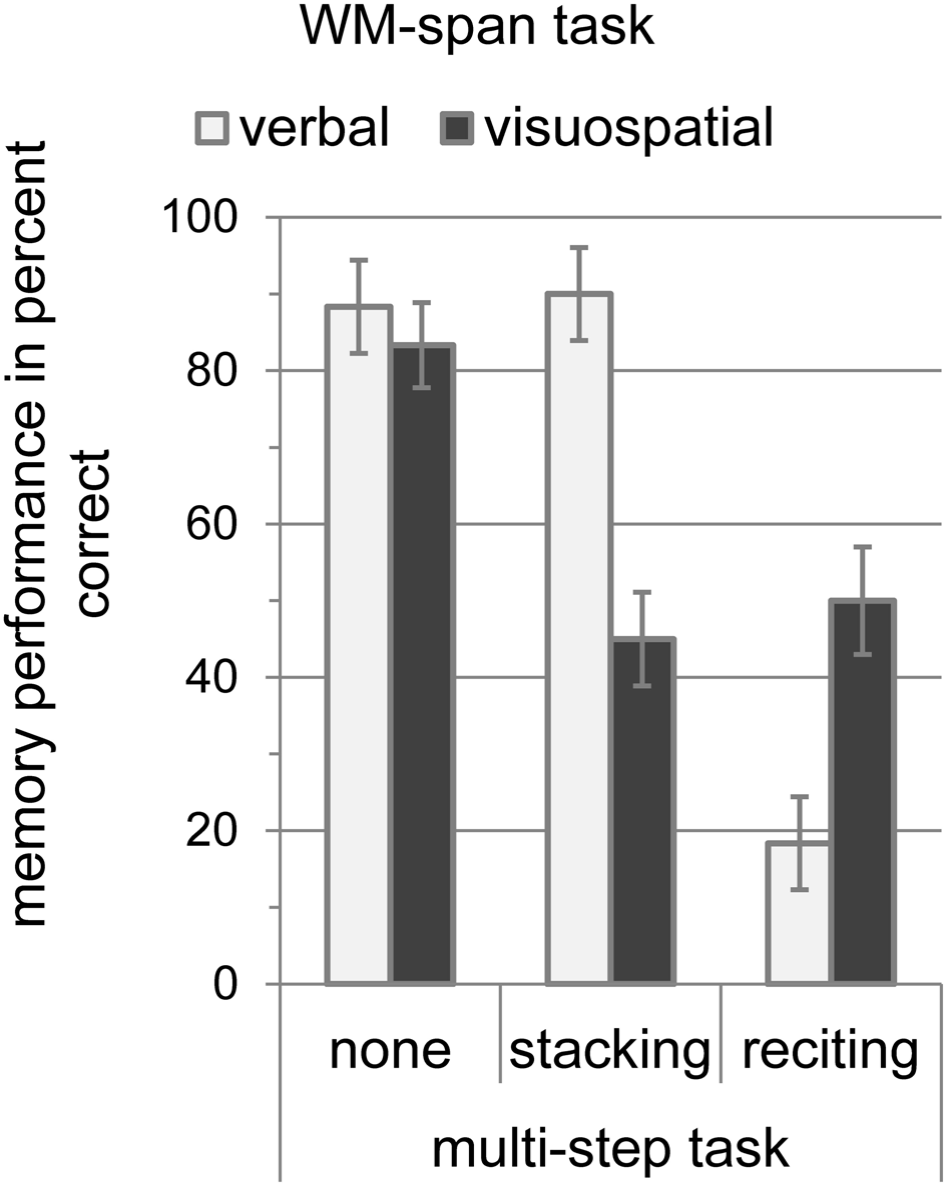
**Memory performance of the WM-span tasks (verbal and visuospatial) in percent correct with standard error of the mean according to Loftus and Masson (1994) during the multi-step tasks (none, stacking, and reciting)**.

Performance measures for the WM-span tasks are depicted in Figure 4. To test whether the multi-step tasks affected the verbal or visuospatial memory span, we conducted a 2 × 3 analysis of variance for the memory performance with WM-span task (verbal and visuospatial) and multi-step task (none, stacking, and reciting) as within-subject variables. The analysis revealed no significant effect of WM-span task [*F*_(1, 9)_ = 0.80, *MSE* = 0.06, *p* > 0.05], indicating that task difficulty was comparable. The main effect of multi-step task was significant [*F*_(2, 18)_ = 51.69, *MSE* = 1.37, *p* < 0.001] with the highest memory accuracy without multi-step task (85.83%), intermediate memory accuracy during stacking (67.50%), and worst memory accuracy during reciting (34.17%). The analysis also revealed a significant interaction between WM-span task and multi-step task [*F*_(2, 18)_ = 24.14, *MSE* = 0.73, *p* < 0.001]. Planned paired *t*-tests with Bonferroni-correction revealed that the verbal WM-span accuracy did not differ significantly between the single task condition (88.33%) and the stacking (90.00%) condition [*t*_(1, 9)_ = 0.36, *SE* = 0.05, *p* > 0.05], while it decreased significantly from 88.33% without dual task to 18.33% with simultaneous reciting [*t*_(1, 9)_ = 7.87, *SE* = 0.28, *p* < 0.001]. The visuospatial WM-span accuracy was reduced significantly from 83.33% without dual task to 45.00% in the stacking condition [*t*_(1, 9)_ = 4.64, *SE* = 0.08, *p* < 0.01], and also decreased significantly from 83.33% without dual task to 50.00% in the reciting condition [*t*_(1, 9)_ = 4.05, *SE* = 0.08, *p* < 0.05]. However, this cross-domain interference between the visuospatial WM span and reciting was significantly smaller than the domain-specific interference between reciting and the verbal WM span [*t*_(1, 9)_ = 2.80, *SE* = 0.13, *p* < 0.05].

## Discussion

The present study asked whether LTM-based attention selection—that is involved in the execution of highly practiced multi-step tasks (Foerster et al., 2011, 2012)—requires WM or can bypass WM. On the one hand, studies on eye movement control in multi-step real-world tasks (e.g., Hayhoe et al., 2003; Droll et al., 2005; Land and Tatler, 2009) point to little use of WM during the execution of such tasks. Moreover, research with highly controlled laboratory tasks on visual search indicates that constant search-target templates are maintained in LTM in a WM-interference free manner (Woodman et al., 2013). On the other hand, selective attention is strongly related to WM (e.g., Olivers et al., 2011). Therefore, it is unclear whether LTM-based attentional selection during the execution of well-learned multi-step tasks requires WM or can bypass WM. We investigated this question on the basis of a WM dual-task paradigm with highly practiced multi-step tasks. Participants were asked to maintain either verbal (letters) or visuospatial (locations) material in WM while they had to perform a highly practiced LTM-based multi-step task that was either a verbal (high-speed poem reciting) or a visuospatial (speed stacking) task.

Results revealed that interference between WM and multi-step tasks was mainly domain-specific. Speed stacking disturbed the visuospatial, but not the verbal memory performance, while poem reciting disturbed the verbal WM performance significantly stronger than the visuospatial WM performance. Moreover, high-speed poem reciting was in general faster and more accurate than speed stacking. Neither reciting nor stacking was affected by the WM-span tasks. The fact that the highly practiced multi-step tasks distorted WM performance in a mainly domain-specific manner support the view that LTM-based control of attention requires domain-specific WM processes.

### Implications for theories of automatization and skill proceduralization

The present study revealed interference between WM retention and the execution of automatized tasks, that is, tasks that have been trained up to a level on which no further improvement has been observed. This finding argues against a conceptualization of automatized and controlled processes as was proposed by Schneider and Shiffrin (1977a,b) implying that automatized processes should not interfere with other processes (see also, Neumann, 1984). Furthermore, our data challenge the suggestion that highly trained skills can be performed without recruiting WM (e.g., Fitts and Posner, 1967; Anderson, 1993; Beilock et al., 2002) including the idea that action-relevant parameters are directly specified via LTM information (Neumann, 1984, 1990; Logan, 1988, 1990). Finally, on the basis of our results, it seems difficult to retain a strict segregation of declarative and procedural WM (e.g., Oberauer, 2009, 2010). At least, the assumption that well-practiced procedures do not interfere with parallel retention of declarative material is called in question.

### How WM is involved in LTM-based control of attention

How might LTM-based control of attention in the overlearned multi-step tasks involve WM processes? In speed stacking, the learned information about important locations in the environment might be retrieved from LTM by writing into a visuospatial map of WM. The same visuospatial map might be involved in the attention-based rehearsal of visuospatial material in WM. This assumption is supported by results from the following studies. First, attention seems to be necessary for LTM retrieval (e.g., Wagner et al., 2005; Cabeza et al., 2008). Second, where to attend while performing a highly practiced sensorimotor task is largely controlled by LTM (Foerster et al., 2011, 2012). Third, there is evidence that the maintenance of visuospatial material in WM might be based on visuospatial attention (e.g., Smyth, 1996; Awh et al., 1998, 2000, 2006; Awh and Jonides, 2001; Theeuwes et al., 2011).

Complementary, the same attention processes might be required for retrieving verbal LTM content for poem reciting as well as for the subvocal articulatory process that constitutes verbal rehearsal (Salame and Baddeley, 1982; Baddeley et al., 1984; Awh et al., 1996). Behavioral and neuroimaging studies (e.g., Zhijian and Cowan, 2009; Majerus et al., 2012) showed that attention is involved in verbal short-term retention. Moreover, Wagner et al. (2005) reviewed neuroimaging studies showing that the posterior parietal cortex (PPC)—an important structure for WM (e.g., Funashi et al., 1989; Fiehler et al., 2011)—is also activated during episodic memory retrieval (see also, Cabeza et al., 2008). The authors proposed that the PPC is activated because memory representations have to be attended for retrieval. Therefore, attention for LTM retrieval during the execution of the multi-step tasks may have competed with attention-based rehearsal for the WM-span tasks.

We assume that attention, WM, and LTM interact during the execution of LTM-based multi-step tasks. Task-relevant information is selected from LTM structures by attention-based domain-specific activation in WM. In neurophysiological terms, long-term synaptic weights—LTM—are transferred into short-term continuous firing in neural circuits—WM (Olivers et al., 2011). Importantly, we assume that LTM representations can only be used for action control, if they have been selected by the same attentional mechanisms that also maintain domain-specific information in WM (see also, Schneider, 2013). Consequentially, a tight interaction should exist between attention, domain-specific WM, and LTM processes during the execution of highly practiced multi-step tasks.

### Further findings: cross-domain interference and asymmetry of interference effects

Two further important findings of our study should be discussed. We start with the question, why poem reciting did not only reduce the verbal WM span, but also the visuospatial WM span, although to a smaller degree. In Baddeley's WM model (Baddeley and Hitch, 1974, 1994; Baddeley, 1986, 2003, 2012), such cross-domain interference can either be due to global WM load (within the central executive and the episodic buffer) or to interference within the visuospatial sketchpad, or the articulatory loop. Global WM load refers to the involvement of the central executive and the episodic buffer, so that tasks compete for processes within these multidimensional WM domains. Global WM load might be higher during poem reciting than during speed stacking. What justifies this assumption? When performing a sensorimotor task in the real world, humans usually gather visual (-spatial) information just when it is needed to perform a sub-action (Hayhoe et al., 2003). This phenomenon has also been observed in speed stacking (Foerster et al., 2011, 2012). This strategy of using the “world as external memory” (O'Regan, 1992) reduces WM load. During high-speed poem reciting, outsourcing of relevant information to the environment is not possible. Information for action programming and execution stems from LTM only. This higher LTM “load” may cause a higher WM load during reciting than during stacking.

However, it is also possible that the observed interference between reciting and visuospatial WM was due to specific interference within the visuospatial sketchpad. Poem reciting itself might imply visuospatial processing. A visual imagery process of words during reciting could have been introduced because of the visual presentation of the poem during initial learning. If participants imagined words while reciting, these words should take limited visuospatial attentional capacity (e.g., attentional weights, Bundesen, 1990) away from attentional selection of information for the multi-step task.

An additional supplementary question is why the interference effects between WM spans and multi-step tasks were unidirectional. While the WM retention suffered from the concurrent execution of the multi-step LTM-driven tasks, these tasks were unaffected by the simultaneous maintenance of information in WM. Participants seem to have prioritized the multi-step tasks over the WM tasks, so that they could maintain at least the performance level of the multi-step tasks to the disadvantage of the WM-span tasks. Future work has to investigate whether explicitly instructing participants to prioritize one task over the other changes the directionality of the interference.

## Summary

The current study has demonstrated first that visuospatial, but not verbal WM was disturbed by an LTM-based multi-step sensorimotor task. Second, verbal WM was affected by a verbal LTM-based multi-step task more than visuospatial WM. Moreover, the two multi-step tasks were not disturbed by concurrent retention of domain-specific information in WM. This finding of unidirectional and mainly domain-specific interference points to a requirement of the same domain-specific attentional mechanism during WM retention as well as during the execution of LTM-based multi-step tasks. Task-relevant information is selected from LTM structures by attention-based domain-specific activation in WM.

### Conflict of interest statement

The authors declare that the research was conducted in the absence of any commercial or financial relationships that could be construed as a potential conflict of interest.
