# Supplementary material for: Long-term memory-based control of attention in multi-step tasks requires working memory: evidence from domain-specific interference
Source: Front Psychol. 2014 May 9;5:408. doi: 10.3389/fpsyg.2014.00408 (PMC4023044; doi:10.3389/fpsyg.2014.00408)
Supplement: Supplementary file 1 [file DataSheet1.DOCX]

1. **Appendix**

The German poem and its English translation.

Nun bin ich hier und soll

find ich es nun ganz toll

will ich mich nun beklagen

ein Kurzgedicht aufsagen

Man hat mich nicht gefragt

ob ich Gedichte mag

ob ich Gedichte hasse

ob ich mich drauf einlasse

Ich halte tapfer aus

auch ohne Standapplaus

ich werd‘s zu Ende bringen

die Sache schon bezwingen

Nun folgt der letzte Part

mir bleibt auch nichts erspart

nun soll ich auch noch sagen

ich hab es gut ertragen

Now here I am and shall

do I find it very nice

I want to complain

and recite a short poem

No one asked me

whether I like poems

whether I hate poems

whether I take up with them

I hold out bravely

not longing for standing ovations

I will bring it to an end

and conquer the thing

Now the final part is come

and I am not spared a thing

now I shall even say

I have taken it well
